# Vehicle Aerodynamic Noise: A Systematic Review of Mechanisms, Simulation Methods, and Bio-Inspired Mitigation Strategies

**DOI:** 10.3390/biomimetics11020099

**Published:** 2026-02-02

**Authors:** Tao Zou, Yifeng Fu, Pan Cao

**Affiliations:** 1 School of Automotive and Traffic Engineering, Jiangsu University, Zhenjiang 212013, China; 2222404008@stmail.ujs.edu.cn; 2 State Key Laboratory of Special Materials Surface Engineering, China Academy of Machinery Wuhan Research Institute of Materials Protection Co., Ltd., Wuhan 430030, China; 3 College of Mechanical Engineering, Yangzhou University, Yangzhou 225127, China

**Keywords:** bio-inspired flow control, bionic vehicle design and optimization, vehicle aerodynamic noise, numerical simulation

## Abstract

With the electrification of automotive powertrains, aerodynamic noise has emerged as the primary factor affecting vehicle comfort. This systematic review, adhering to PRISMA 2020 guidelines, bridges the gap between biological fluid mechanics and automotive engineering by synthesizing recent advances in aerodynamic mechanisms and bionic control strategies. Based on a comprehensive search of Web of Science, ScienceDirect, SAE Mobilus, and Google Scholar for the literature published between 2016 and 2025, 90 eligible studies were analyzed to provide a rigorous evidence-based synthesis. The review details complex flow phenomena, such as turbulent separation and vortex shedding across key regions like A-pillars and mirrors, drawing parallels to bio-inspired fluid–structure interactions. Numerical prediction methods, including large eddy simulation (LES), detached eddy simulation (DES), and lattice boltzmann method (LBM), are critically examined for their efficacy in resolving both conventional and bionic flow structures. A significant focus is placed on bio-inspired mitigation technologies, where quantitative findings demonstrate substantial noise suppression: specifically, the reviewed data shows that bionic riblet surfaces on tires can reduce noise levels by up to 5.18 dB, while beetle-head-inspired protuberances on exterior mirrors can achieve reductions of up to 10 dB. Finally, this work suggests future research directions in integrated fluid–acoustic–structural simulation frameworks and self-adaptive bionic systems, providing a robust reference for developing high-performance, low-noise vehicles inspired by natural organisms.

## 1. Introduction

With the electrification of automotive powertrains and the marked reduction in conventional noise sources like engines, aerodynamic noise has rapidly become the dominant in-cabin noise source at high speed, severely compromising overall noise, vibration, and harshness (NVH) performance and occupant comfort [1,2,3]. Aerodynamic noise generally becomes the dominant interior sound source once speeds surpass approximately 100 km∙h^−1^, although this transition occurs notably earlier for bluff-bodied vehicles like SUVs due to their higher drag coefficients. At this stage, turbulent boundary-layer separation, vortex interactions, and minute leakage gaps in sealing structures excite broadband wind noise; once transmitted into the cabin, the noise not only degrades speech intelligibility and sound-quality perception but can also induce auditory fatigue and driver distraction, thereby jeopardizing road safety [4,5]. The impact is particularly pronounced in battery-electric and autonomous-driving scenarios, where the absence of masking the powertrain noise renders aerodynamic noise more readily perceptible and subjectively disturbing [3,6]. Mechanistically, the sources are diverse: dipole and quadrupole source noise induced by structures such as the A-pillar and exterior mirror, monopole-type leakage noise arising from weak sealing regions around doors and side windows, and cavity resonance and buffeting phenomena, all contribute to the overall sound field [7]. In addition, the high-frequency content of wind noise readily couples with flexible panels such as windows, undermining sound insulation and constraining lightweight design [8]. Bio-inspired mitigation technologies, such as surfaces modeled after owl feathers and shark skin, disrupt large-scale vortex coherence to suppress noise. For instance, Wang et al. [9] reported that the implementation of bio-inspired flexible flaps can reduce radiated sound power by up to 60.3% through the effective suppression of lift oscillations, demonstrating the high efficiency of passive fluid–structure interaction in aeroacoustic control. This field has further evolved to encompass advanced biological principles—including flexibility, flapping kinematics, fish mucus mimicry, and autonomous feedback strategies [10]—to achieve more sophisticated and adaptive flow modulation. Traditional reliance on wind-tunnel and road tests imposes severe limitations in terms of time, cost and early-stage predictability, making it difficult to meet the dual demand for efficiency and accuracy in modern vehicle development [11]. With the maturation of numerical techniques such as computational fluid dynamics (CFD), computational aeroacoustics (CAA) and statistical energy analysis (SEA), virtual modeling of source identification, path analysis and structural optimization has become feasible; in particular, Large Eddy Simulation (LES), hybrid CAA frameworks and the boundary-element method (BEM) have evolved into core tools for wind-noise research, providing efficient and systematic support for whole-vehicle development [3,6,12]. Furthermore, emerging frameworks such as the frequency-domain spectral element method (SEM) offer high-order accuracy for modeling complex heterogeneous media, pointing toward new avenues for aeroacoustic research [13]. Consequently, a systematic understanding of the generation mechanisms, transmission paths and psycho-acoustic perception of automotive wind noise, and the construction of an integrated multi-scale simulation and control framework, is of great practical significance for enhancing vehicle comfort and perceived quality while underpinning the development of intelligent, low-carbon transport systems and is a key enabler for meeting broader transportation sustainability goals, such as the reduction in greenhouse gas emissions [14].

Aerodynamic noise as the principal noise source under high-speed conditions can be traced back to early studies in the 1960s. Early studies focused on monopole-dominated leakage noise arising from door gaps and imperfect window seals. With advances in sealing technology, such noise has declined markedly, and research attention has progressively shifted to dipole-type wind noise generated by turbulent separation around the A-pillar, exterior mirrors, and similar structures [15]. Since the 21st century, the advent of CFD and CAA has driven a paradigm shift from wind-tunnel testing towards simulation-based prediction, and numerical analysis now plays an indispensable role in source identification and sound-field propagation studies [16]. Researchers have also introduced psycho-acoustic metrics such as loudness, sharpness, and fluctuation strength to quantify the perceptual impact of wind noise on occupants [17]. In recent years, propelled by electric vehicles and autonomous driving, conventional passive countermeasures face new challenges, while active noise control (ANC), biomimetic structures, and other emerging technologies are being progressively adopted in vehicle development. The transition to electric mobility has fundamentally altered the vehicle acoustic landscape. Without the masking effect of the internal combustion engine, aerodynamic noise has become the dominant noise source at significantly lower speeds. This shift has necessitated a parallel evolution in research focus: moving from traditional sealing mechanisms to high-fidelity numerical simulation and emerging active control strategies. To address this evolving technical landscape, this review is organized into three core domains: Section 3.1 details the generation mechanisms of these increasingly prominent sources; Section 3.2 critically examines the numerical methods required to capture them; and Section 3.3 evaluates advanced control technologies, highlighting the transition from passive to active and bio-inspired solutions.

This review addresses the key scientific questions and engineering challenges of vehicle aerodynamic noise by systematically summarizing advances in generation mechanisms, numerical simulation methods and state-of-the-art control technologies. First, it outlines the fundamental physical mechanisms and typical source regions of aerodynamic noise, analyzing their characteristic manifestations in the A-pillar and side-window area, exterior mirror, tires and underbody, and wake vortex region. Next, it reviews mainstream numerical methods developed in recent years, including flow-field modeling, acoustic prediction, and CFD–CAA coupling strategies. The article then synthesizes a wide range of control techniques, from body-shape optimization and sound-absorbing/damping materials to ANC and biomimetic flow manipulation, before concluding with a summary and prospects. The aim is to provide a systematic reference and trend assessment for researchers and engineers that are engaged in wind-noise studies and vehicle development.

## 2. Methods

This review follows the PRISMA 2020 guidelines: a framework that promotes transparent reporting of a systematic review’s purpose, procedures, and findings. The completed PRISMA 2020 checklist is provided in the Supplementary Materials (Table S1). Adhering to this standard ensured a rigorous, reproducible search and selection process [18,19]. Because the work involves neither clinical interventions nor patient data, prospective registration was not required.

### 2.1. Search Strategy

**Figure 1:**
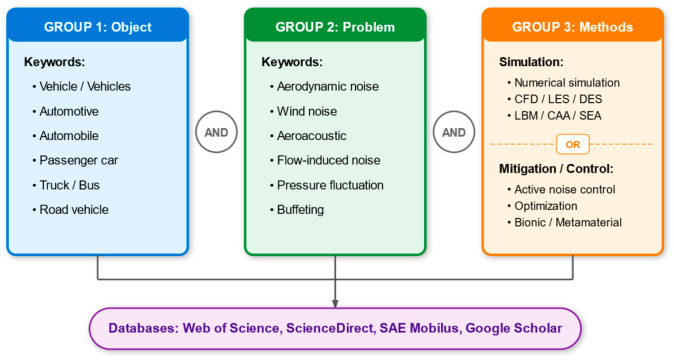
Search keywords and strategy framework for the systematic review on vehicle aerodynamic noise.

A Boolean query encompassing three concept blocks—(road vehicle), (aerodynamic noise), and (simulation/mitigation technology)—was executed in four databases (Web of Science Core Collection, ScienceDirect, SAE Mobilus, and Google Scholar) on 20 June 2025. These specific databases were selected to ensure a balanced coverage of high-impact academic journals (Web of Science, ScienceDirect), industry-standard engineering reports (SAE Mobilus), and broad gray literature (Google Scholar), thereby minimizing publication bias and ensuring both theoretical and practical developments were captured. An illustrative Web of Science syntax is as follows: “ALL = (vehicle OR car* OR truck* OR bus* OR “road vehicle”) AND ALL = (“aerodynamic noise” OR “wind noise” OR aeroacoustic*) AND (ALL = (CFD OR LES OR URANS OR DES OR LBM OR CAA OR BEM) OR ALL = (“active noise control” OR ANC OR “plasma actuator” OR “acoustic metamaterial” OR “flow-control device” OR “shape optimization”))”. Search keywords and the strategy framework for the systematic review on vehicle aerodynamic noise are shown in Figure 1.

### 2.2. Configure Exclusion and Inclusion Criteria

To ensure methodological rigor and relevance to the review objectives, a set of predefined inclusion and exclusion criteria was developed based on the publication timeframe, document type, research content, and vehicle scope. Only peer-reviewed journal articles published between 1 January 2016 and 20 June 2025 were considered. The selection of this timeframe ensures that the review captures the most significant technological shifts following the rapid electrification of powertrains, while the focus on peer-reviewed journals prioritizes the high reliability and technical maturity of the synthesized data. Eligible studies were required to focus on either (i) numerical aeroacoustic simulation, including methods such as CFD, LES, CAA, BEM, or hybrid approaches; or (ii) advanced aerodynamic noise mitigation technologies, such as ANC, plasma actuation, acoustic metamaterials, flow-control devices, or shape optimization. Studies that lacked a clear focus on either simulation or mitigation were excluded. Furthermore, to ensure the rigor and technical depth of this review, a stringent definition of “irrelevance” was applied. Studies were excluded if they did not pertain to road vehicles—specifically excluding aircraft, trains, motorcycles, UAVs, and eVTOLs—or if they failed to focus on high-fidelity numerical aeroacoustic simulations (e.g., CFD, LES, BEM) and advanced noise mitigation technologies (e.g., ANC, bionic structures, or shape optimization). This refined categorization ensures that only research that directly contributed to the core objectives of automotive wind noise control was retained. This specialized scope addresses the unique fluid–acoustic coupling mechanisms and boundary conditions—such as ground effects and rotating wheel flows—that characterize road vehicles. While this ensures a deep analysis of the primary subject, the authors acknowledge that the exclusion of other transport sectors may limit the exploration of cross-industry innovation opportunities, which remains a valuable direction for future interdisciplinary research. These criteria are summarized in Table 1. The screening process was conducted in two stages. In the first stage, titles and abstracts were screened to eliminate clearly irrelevant studies. In the second stage, full-text articles were reviewed for final eligibility. Screening was independently performed by two reviewers, and discrepancies were resolved through consensus or consultation with a third reviewer.

## 3. Results and Discussion

**Figure 2:**
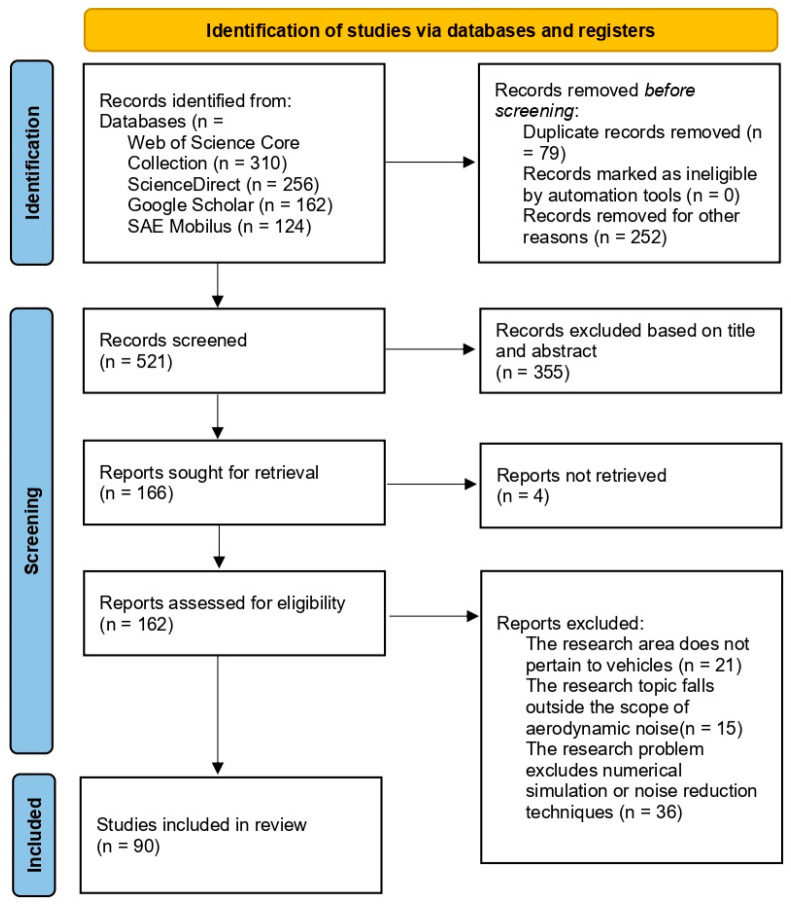
PRISMA 2020 flow diagram, showing identification, screening, and inclusion of studies [18].

A total of 852 records were identified through searches in four databases: Web of Science Core Collection, ScienceDirect, SAE Mobilus, and Google Scholar. After removing 79 duplicate records and 252 records outside the specified timeframe or document type, 521 records remained for title and abstract screening. During this stage, 355 records were excluded because they focused on non-road transport systems—including aircraft, high-speed rail, and heavy agricultural machinery—or lacked a clear emphasis on high-fidelity numerical aeroacoustic simulation and flow control. The full texts of 166 articles were sought, and 162 were successfully retrieved. Following the full-text review, 72 articles were excluded for specific technical reasons: 21 records had a non-vehicle focus, 15 were outside the specific research scope of aerodynamic noise by focusing solely on steady-state drag or thermal management, and 36 lacked a detailed simulation or mitigation methodology. Ultimately, 90 high-quality studies met all inclusion criteria and were included in the final review. The full selection process is illustrated in the PRISMA 2020 flow diagram in Figure 2. At the same time, to provide a structured overview, these studies are categorized and summarized based on their primary focus, including bio-inspired strategies, aerodynamic mechanisms, numerical methodologies, and other related aspects (see Appendix A, Table A1, Table A2, Table A3 and Table A4 for a summary of all included studies).

### 3.1. Generation Mechanisms and Characterization of Aerodynamic Noise

#### 3.1.1. Mechanisms and Classification of Aerodynamic Noise

Aerodynamic noise is generated when the unsteady aerodynamic forces and vortex-induced pressure fields that develop around a moving vehicle perturb the surrounding air and radiate compressive waves. Building on the aero-acoustic analogy first proposed by Lighthill [20] and subsequently generalized for boundary interactions and shear-layer effects by Ffowcs Williams–Hawkings (FW-H) [21,22], the turbulent flow field can be idealized as a superposition of three canonical sources—monopole, dipole and quadrupole—whose mechanisms, typical on-car locations, and relationship between sound power (I/dB) and flow velocity (v/m∙s^−1^) are summarized in Table 2. Dipole sources, driven by fluctuating surface pressures on protruding edges such as the A-pillar, exterior mirror, and wheel arch, dominate the mid-frequency content that reaches the cabin, whereas quadrupole sources associated with free-shear turbulence in the underbody and wake regions gain prominence with increasing speed. Monopole sources, linked to periodic volumetric pulsation at leakage paths in door-seal gaps or body joints, are usually secondary, provided sealing integrity is maintained.

Automotive aerodynamic noise thus originates from a combination of turbulent excitation, shear-layer vortex shedding, leakage through structural gaps, and cavity resonance phenomena [23,24]. In engineering practice, these physical processes are grouped into four functional classes—pulsation noise, suction/leakage noise, cavity noise, and buffeting-induced panel vibration—each of which can be mapped to the monopole–dipole–quadrupole triad, and together they furnish the theoretical basis for the detailed source-region analysis that follows [1,25].

#### 3.1.2. Typical Aerodynamic Noise Source Regions and Influencing Factors

**Figure 3:**
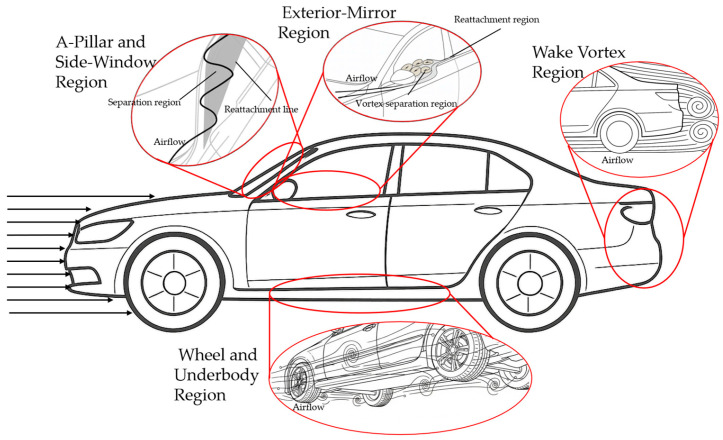
Schematic illustration of the four principal aerodynamic noise source regions on a passenger car.

Under the action of high-speed airflow, vehicle body shapes and ancillary structures induce complex flow separation and vortex structures; these local flow phenomena constitute important sources of typical aerodynamic noise. Because different regions differ markedly in structural morphology, relative flow velocity, and spatial proximity to occupants’ ears, their sound-radiation characteristics and dominant noise mechanisms likewise vary. For example, as depicted in Figure 3, the A-pillar and side-window region, located close to the cockpit and characterized by pronounced flow separation, often forms strong dipole sources. The exterior-mirror region, a protuberant structure, tends to generate broadband noise and whistle tones due to vortex shedding from its housing, stalk, and gaps. The tire and underbody region, by virtue of its geometrical complexity, readily intensifies flow separation, and in the wake vortex region, the unstable coalescence of high-speed trailing flow can cause large-scale vortical structures to strike the rear deck or the surfaces around the tail lamps periodically, significantly affecting the sound-field distribution in the rear cabin [26]. It is crucial to note that the hierarchy of these sources evolves with powertrain electrification. While the A-pillar and mirrors remain the dominant high-frequency dipole sources for internal combustion engine (ICE) vehicles, the absence of masking engine noise in electric vehicles (EVs) has elevated the prominence of low-frequency contributions from the underbody and wheelhouse regions. In view of this shift, the following subsections will systematically discuss the aerodynamic features, flow structures, and their mechanisms of influence on in-car wind noise for each of these key regions, providing a foundational basis for acoustic modeling and control design.

##### A-Pillar and Side-Window Region

The A-pillar and side-window zone is a major aerodynamic noise source, due to flow-acoustic coupling. At the A-pillar, a sharp geometric turn causes 3D flow separation, forming a conical vortex along the window. Interaction with the mirror wake generates broadband noise, escalating with vortex evolution. An open side window makes the compartment act as a cavity; shed vortices break at the edge, inducing pressure pulsations via two mechanisms. Downstream-convecting vortices striking the rear edge create upstream pressure waves, forming an acoustic feedback loop, and vortex-shedding frequency matching the cavity’s natural frequency triggers Helmholtz resonance.

Solutions therefore require combined flow and acoustic optimization. Reshaping the A-pillar suppresses the separation strength, while non-smooth surfaces disrupt large vortex coherence. Side-window deflectors delay vortex formation and shorten its path, reducing pressure pulsations. Spatial variability is key: the front window has lower noise than the rear due to shorter airflow paths and smaller vortex scales, while the mirror position affects the shear-layer stability and mid-high frequency noise spread. Effective suppression balances vortex control with resonance interruption, necessitating co-optimization of the A-pillar angle, mirror layout, and deflector geometry [27,28].

##### Exterior-Mirror Region

The exterior mirror is a major vehicle aerodynamic noise source, especially at high speeds. Noise primarily stems from flow separation over the mirror housing and vortex shedding in its wake. Airflow past the mirror creates pressure fluctuations and turbulence, generating significant noise that compromises acoustic targets [29,30].

Mirror geometry and position directly impact noise. Studies confirm that the mirror-window gap, base position, and mounting angle alter the flow structure, affecting the noise levels. For example, inclined (vs. horizontal) mounting cuts noise by altering body vortices [31,32]. Additionally, the mirror-A-pillar positioning influences vortex strength and mirror-wake interactions with the side window, making it critical for noise control [33,34,35].

##### Wheel and Underbody Region

Non-pneumatic tires (NPTs) with open spokes induce complex flow separation during high-speed rotation, generating intense vortex noise. Studies show that airflow cutting through spokes produces strong vortex cores in gaps and near the hub; the Lamb vector quantifies the source strength, with the vortex energy peaking in the 120–270° contact sector. Biomimetic riblet surfaces (e.g., triangular ribs) on spokes suppress large-scale vortices, reducing the Lamb vector magnitude and disrupting vortex bands, achieving up to 5.18 dB noise reduction. This occurs by fragmenting vortices into smaller structures, lowering the fluid strain energy and acoustic efficiency [36].

Underbody flow-separation noise dominates electric vehicle cabin acoustics. Complex underbody geometry (motors, wheel arches, etc.) amplifies separation, causing strong 100–300 Hz pressure pulsations near floor panels. Noise enters via turbulent pressure (fluid–structure coupling) and acoustic pressure (acoustic-structure coupling); turbulent pressure contributes more, due to convective wavenumber concentration. Vortex strikes on panels create directional dipole sources, with 5 dB higher rear sound-pressure level (SPL) than the front. Deflectors lower the wheel turbulence, while side skirts attenuate low-frequency pulsations by reducing battery-area separation [37]. Increasing panel thickness improves transmission loss but requires avoiding resonance from panel-cavity mode coupling [38].

##### Wake Vortex Region

The wake is a major source of vehicle aerodynamic noise, with vortex shedding and re-attachment shaping the noise spectrum. Studies show that rear spoiler parameters—inclination (α), length (L), and diffuser angle (θ)—strongly control the wake: increasing θ to 30° while setting boat-tail angle β to 0° streamlines flow, reduces vortex core strength by ~20%, and lowers sound-power by 14 dB(A). This stems from suppressed separation at the diffuser, diminishing quadrupole source radiation. Longer spoilers reduce pressure fluctuations but risk shifting shedding frequencies to excite resonances if overextended [39].

Vortical system interference complicates mechanisms. Side-mirror horseshoe vortices (V_1_, V_2_) interacting with the A-pillar vortex create asymmetric wake vortices. When the vorticity difference exceeds 5.4%, spatial instability arises, producing spectral peaks. Tilting the mirror to 16° optimizes the vortex layout, reducing interference noise by 10 dB despite a potential 7.3% drag increase. Vortex breakdown at the rear window’s lower edge also excites 30–80 Hz pulsations; reinforcing the seal can cut transmitted noise by 3–5 dB due to boundary-condition sensitivity [40].

##### Other Noise Sources

Beyond primary sources, vehicles have secondary aerodynamic noise mechanisms. Sealing-system noise occurs when door weather strips vibrate under high-speed flow, with the material viscoelasticity and cross-section governing transmission loss, creating particularly weak zones at 1–3 kHz [41]. Auxiliary-equipment noise arises from high-speed rotating machinery: fuel-cell compressors (~90,000 rpm) excite “buzz-saw” harmonics [42], while alternator fans emit blade-pass-frequency harmonics (4th–18th order) [43].

Sharp structural edges induce distinct noises: engine-hood kinks trigger self-excited feedback loops, causing narrow-band whistles [44], and serrated fan edges redistribute vortex energy into broadband noise [45]. These are highly geometry-sensitive.

Structural-resonance noise involves fluid–solid coupling. Vortex interference in fan tip clearances (~5 mm) intensifies with rotational speed [46], while sealing-cavity modal vibrations degrade acoustic performance in specific bands [41]. Though 10–15 dB lower than primary sources, secondary noises occupy frequency ranges that are critical for human hearing and impact NVH refinement [45,47].

#### 3.1.3. Evaluation Metrics and Objective–Subjective Characterization of Aerodynamic Noise

The evaluation framework for aerodynamic noise is evolving from the single metric of SPL to a multi-dimensional description that combines objective and subjective indicators. Objective evaluation: Researchers commonly use wavenumber-frequency decomposition to separate hydrodynamic pressure from acoustic pressure, and they quantify their differing transmission efficiencies through body structures with the metric of transmission loss (TL). It has been shown that the “modal-preference effect” and “resonance effect” of side-window glass make it a low-wavenumber filter, so hydrodynamic-pressure energy decays by more than 25 dB compared with acoustic-pressure energy—this is the core mechanism underlying the transmission gap between the two components [48]. The recently developed dynamic noise transfer function (NTF) model represents noise sources uniformly as “forces” acting on the body; when combined with wall-pressure fluctuations extracted by CFD, it can quantify vehicle sensitivity to noise transmission more accurately [49].

In the subjective evaluation domain, psycho-acoustic parameters serve as the essential bridge between physical metrics and human auditory perception. Zwicker loudness (loudness), sharpness (sharpness), roughness (roughness), and fluctuation strength (fluctuation strength) are widely used to assess the in-cabin sound quality. Their predictive power in steady wind noise is summarized in Table 3. Experiments show a pronounced non-linear relationship between conventional SPL and subjective perception: when SPL exceeds 50 dB(A), each 1 sone increase in loudness is equivalent to a 0.036–0.038 acum increase in sharpness in terms of its negative impact on acceptance [50]. For non-stationary gusting noise, Carr and Davies [51] proposed the revised gusting metric, ($G_{rev}$), which synthesizes modulation depth, duration, and rate. Its calculation model is as follows:

(1) $$G_{rev}=\sum_{n}\frac{\Delta N_{n}\cdot T_{n}}{\frac{4}{f_{mod\left(n\right)}}+\frac{f_{mod\left(n\right)}}{4}}$$

This model can effectively predict auditory annoyance induced by high-speed turbulence: a reduction of 0.72–0.73 vacil in the index corresponds to the same subjective relief as a 1 sone decrease in loudness. It is worth noting that, because powertrain noise is absent in electric vehicles, the sharpness component of aerodynamic noise becomes more salient, and sound quality above 250 Hz gains markedly higher weight in ride comfort [52].

Integration of objective and subjective characterization is a current research frontier. On one hand, acoustic transfer path analysis (ATPA) combines fluid excitations extracted by CFD with finite-element (FEM) simulations to predict sound radiation caused by structural vibration [53]. Psychoacoustic metrics provide deeper insight into subjective annoyance. Fluctuation strength, for instance, quantifies the perception of slow amplitude modulation. It peaks at a modulation frequency of 4 Hz—which represents the rate of temporal variation, rather than the audible carrier pitch—making it critical for evaluating low-frequency buffeting [54]. Conversely, for stationary wind noise, Carr and Davies [50] identified sharpness (high-frequency spectral balance) as the most significant secondary metric alongside loudness, whereas fluctuation strength showed a weaker correlation in their stationary flow experiments. Future work needs to explore the spatio-temporal coupling mechanism of objective–subjective metrics under transient aerodynamic excitation and to determine the suppression limits of high-frequency noise transmission imposed by non-linear deformation of body sealing systems [55,56].

### 3.2. Numerical Simulation Techniques for Aerodynamic Noise

Aerodynamic noise simulation has evolved into a multi-scale, integrated workflow whose guiding principle is to trade off numerical accuracy against computational cost. Contemporary approaches can be grouped into direct and hybrid strategies. Direct methods solve the fully compressible Navier–Stokes equations in a single step, thereby capturing the coupled fluid–acoustic field without modeling assumptions, but they require very large meshes and long run-times. Hybrid methods split the task: an unsteady flow field is first generated with a turbulence-resolving solver such as LES or DES, and the resulting surface pressures (or volumetric source terms) are then injected into an acoustic analogy formulation—typically Lighthill or Ffowcs Williams–Hawkings—to propagate sound to the observer. This two-stage route delivers most of the spectral detail of the direct approach while keeping resource demands acceptable for full-vehicle studies [57]. Recent practice has converged on three standardization fronts. Mesh design now combines an unstructured core with prismatic boundary-layer cells to achieve adequate near-wall resolution, and uses targeted refinement in vortex-shedding regions to preserve energy-containing scales. The boundary conditions are prescribed in a wind-tunnel-consistent manner: inlet turbulence levels and hydraulic diameters are matched to the chosen Reynolds number, and a slip wall or moving-ground model suppresses artificial shear on the road plane. Discretization schemes favor second-order spatial accuracy; pressure–velocity coupling is handled with SIMPLE in steady sub-tasks and PISO in transient ones. Validation studies show that coupling a low-Reynolds-number eddy-viscosity model with a scale-adaptive treatment can capture the dominant vortex-induced sound components while retaining good fidelity in global aerodynamic loads, confirming the practical reliability of this integrated framework [58,59]. To highlight the practical choices available for vehicle aeroacoustic work, Table 4 collates the mainstream numerical routes, distilling their core workflow, typical merits, and drawbacks.

#### 3.2.1. Flow-Field Simulation Technology

Numerical prediction of vehicle aerodynamic noise relies on high-accuracy flow-field solvers whose essence is to capture turbulent structures and pressure fluctuations precisely. At present, the mainstream approaches center on three turbulence-modeling frameworks—DES, LES, and the Reynolds-averaged Navier–Stokes (RANS) equations—supplemented by hybrid algorithms and high-efficiency numerical discretization strategies to balance computational accuracy with resource consumption.

##### Mainstream Turbulence Models

DES combines the boundary-layer treatment capability of RANS with the transient-vortex-resolution strength of LES and has therefore become a routine tool for external vehicle-flow simulations [65]. Improved variants—such as the delayed detached eddy simulation (DDES) and stress-blended eddy simulation (SBES)—further optimize the RANS/LES switching mechanism, effectively suppress grid-induced separation, and are suitable for transient-flow prediction around complex geometries (e.g., exterior mirrors and A-pillars) [58,66,67]. LES directly resolves large-scale turbulent structures while modeling the sub-grid scales with appropriate sub-grid-scale (SGS) closures; although highly accurate, its computational cost is substantial, so it is typically reserved for fine-scale simulations of local key regions, such as the side-window separation zone [68,69]. RANS models (e.g., the Realizable k-ε and SST k-ω) remain widely used for steady-state initialization and parametric studies owing to their high efficiency, but their ability to predict strong separation and unsteady phenomena is limited [70,71]. The selection of numerical methods for micro-scale bionic structures depends largely on the Reynolds number range and the required fidelity. For bionic features involved in near-wall turbulence control, such as riblets or fins, accurately resolving the interaction between the boundary layer and geometric features is critical. In high-Reynolds-number scenarios, wall-resolved large eddy simulation (WRLES) combined with acoustic analogies has become a recognized standard for capturing high-frequency noise reduction. Conversely, for lower Reynolds numbers that are typical of micro-aerial applications, simplified unsteady models offer a more computationally efficient alternative by capturing non-steady loading without the full overhead of LES. For preliminary design screening where only broadband noise estimates are required, RANS combined with noise source models can provide adequate results at a minimal cost, though it remains unable to resolve detailed unsteady mechanisms.

##### Numerical Discretization and Boundary Treatment

To balance fluid and acoustic accuracy, the governing equations are frequently solved with compressible solvers—such as the lattice Boltzmann method (LBM) or compressible LES [69,72]. Boundary conditions must suppress artificial reflections: non-reflecting boundaries and sponge layers are commonly used to absorb far-field acoustic waves, while wall surfaces employ adaptive boundary-layer meshes (y^+^ ≈ 1) to capture near-wall turbulence accurately [7,11]. Time marching generally uses an implicit second-order scheme, and the time step must meet the acoustic criterion to ensure adequate resolution of high-frequency sound waves [67,71].

##### Mesh Strategy and Computational Optimization

Complex geometries (e.g., full-vehicle models) require multi-level mesh refinement and multi-block structured grids [73]; in acoustic-source regions (the exterior-mirror wake and the A-pillar vortex-shedding zone), local refinement is applied with cell sizes ≤ 2 mm, and the boundary-layer mesh growth rate is kept ≤ 1.2 to limit numerical dissipation [45,66]. Polyhedral meshes and trimmed meshes enhance the discretization efficiency of complex curved surfaces while maintaining accuracy [58,74]. To cut the computational cost, mesh-merging techniques and parallel-computing optimization are widely adopted—for example, mesh stretching is used to soften interface discontinuities and suppress spurious noise, while acoustic-selective damping is introduced to attenuate high-frequency numerical oscillations [36,69]. However, micro-scale bionic simulations impose extreme sensitivities on spatial and temporal resolution. Insufficient resolution often leads to the overestimation of sound pressure levels, making systematic mesh convergence studies particularly vital for these applications. To manage the scale disparity between micro-features and the vehicle body, subdomain simulation strategies are often employed to extract high-fidelity source characteristics that can then be mapped to macro-scale models. The validation gap remains a significant challenge; while aerodynamic coefficients such as pressure distributions are frequently verified, the scarcity of experimental acoustic data for micro-bionic structures limits confidence in predicted noise reduction. Future methodologies should prioritize integrated aero-acoustic validation, comparing predicted noise spectra directly with experimental measurements, rather than relying solely on aerodynamic performance.

#### 3.2.2. Acoustic Simulation Techniques

The acoustic simulation techniques for vehicle aerodynamic noise focus on identifying noise sources, quantifying propagation paths, and verifying control strategies; their core is to capture the fluid–structure acoustic coupling mechanism accurately by numerical means. Present mainstream approaches fall into two categories—hybrid numerical methods and direct numerical computation—supplemented by the source-localization techniques and acoustic-transmission models of sealing systems, thereby forming an integrated prediction framework.

##### Mainstream Numerical Simulation Methods

Hybrid methods predict noise by coupling CFD with acoustic propagation models. Specifically, for low-Mach-number flows, a hybrid strategy that combines the incompressible assumption with aero-acoustic analogies—e.g., the FW-H equation or the acoustic perturbation equation (APE)—can greatly reduce the computational cost [75]; the APE, by separating source terms from propagation terms, efficiently predicts sound-pressure radiation [70,76]. Beyond these analogies, the CFD–BEM (boundary-element method) framework is widely used for aero-acoustic analysis of rotating machinery [77]: Sun et al. [42] extracted blade-surface unsteady pressure from transient-flow calculations and, together with BEM solving the Helmholtz equation, successfully predicted the buzz-saw-noise spectrum of a fuel-cell air compressor. For mid-/high-frequency prediction, the CFD–SEA framework is effective: Oettle et al. [78] employed the LBM to simulate the external flow, quantified door-seal TL via an SEA model, and evaluated wind-noise reduction in door sealing. Zhang et al. [5] further combined LBM with SEA, experimentally calibrating the TL spectrum of the sealing structure and markedly improving interior wind-noise prediction accuracy. The FW-H acoustic analogy, often paired with LES, serves as an efficient tool for far-field noise of components, such as exterior mirrors; e.g., Hamiga et al. [79] used LES data and FW-H integration to resolve the aerodynamic noise directivity of an Ahmed body, confirming the mirror-wake as the dominant source region.

Direct numerical computation (DNC) captures the generation and propagation of sound waves directly by solving the compressible Navier–Stokes equations, thereby avoiding the errors introduced by simplified source models. High-order finite-volume schemes perform especially well at low Mach numbers: Dawi et al. [71] combined a compressible solver with improved delayed detached eddy simulation (IDDES) and, on an SAE vehicle model, directly resolved the link between A-pillar vortex shedding and side-window sound pressure, then separated acoustic- and turbulent-pressure components via wavenumber-frequency analysis. The APE, obtained by filtering vorticity and entropy modes, is dedicated to sound-wave propagation. Guseva et al. [67] built an APE-based wave equation, introduced acoustic damping to suppress spurious noise, and accurately reconstructed the sound field in the exterior-mirror/side-window region. Notably, vortex-sound theory underpins DNC [80]: Zhou et al. [36] quantified vortex-source strength using the Lamb vector and showed that a non-smooth riblet surface suppresses vortex shedding, reducing tire aerodynamic noise by up to 5.18 dB. Building on these frameworks, Rajamuni et al. [81] introduced an immersed boundary-regularized lattice Boltzmann method (LBM), based on a linearized splitting of the weakly compressible Navier–Stokes equations, which significantly enhances the fidelity of modeling fluid–structure–acoustics interactions involving large deformations in complex environments.

##### Source Identification and Localization Techniques

Accurate source localization is a prerequisite to noise control. Independent component analysis (ICA) separates multi-source noise signals: Sun et al. [42] used ICA to decompose electromagnetic noise, rotor-stator tonal noise, and broadband turbulent noise, identifying the dominant component of centrifugal-compressor aerodynamic noise. Wavenumber-frequency analysis excels at distinguishing acoustic from turbulent-pressure components: Dawi et al. [71] applied two-dimensional Fourier transform to identify the acoustic-wavenumber range on an SAE body side-window surface ($\left|k\right|<\omega /c$), whereas the turbulent pressure was concentrated near the convective wavenumber (k≈ω/U).

##### Acoustic-Transmission Modeling of Sealing Systems

Door and window-frame sealing strongly influence interior noise. SEA quantifies seal sound-insulation by defining a TL spectrum: Zhang et al. [5] discretized seals into TL panels and coupled them to an SEA cabin model, finding window-frame seals to dominate mid-/high-frequency interior noise. For complex seals, a finite-element/SEA hybrid model (FE–SEA) is suitable: Deng et al. [82] predicted door-seal transmission loss with this model and validated high-frequency accuracy experimentally. Seal-cavity resonance may amplify noise transmission: Oettle et al. [78] reported that shear-layer oscillations inside door-seal cavities can excite acoustic modes and boost specific frequency bands.

#### 3.2.3. Validation and Uncertainty Analysis of Numerical Simulations

In vehicle aerodynamic noise research, the reliability of numerical simulation depends critically on a rigorous validation workflow and a systematic investigation of uncertainty sources. Existing studies show that validation is generally achieved by comparing numerical results with experimental data, covering both flow-field characteristics and acoustic performance. Li et al. [83] verified a CFD model of an intake system against steady pressure-loss measurements, and further confirmed the accuracy of a finite-element acoustic model using noise reduction (NR) tests in a semi-anechoic chamber; the errors were within engineering limits (static-pressure-loss deviation ≤ 9.1%, spectral trend agreement). Likewise, Wang et al. [84] compared surface-pressure fluctuations on an exterior mirror with far-field sound-pressure levels in a wind tunnel and showed that an LES coupled with the Lighthill acoustic analogy (LAA) predicted low-frequency buffeting noise with errors below 2%, demonstrating the engineering suitability of the hybrid method. The main sources of uncertainty are as follows:

##### Choice of Computational Models and Algorithms

Different turbulence models (e.g., URANS, LES, DES) yield markedly different accuracies in capturing vortex structures [85], thereby affecting source-noise prediction. Broatch et al. [86] pointed out that in turbine-compressor aerodynamic noise simulations, LES captures near-stall rotating-stall phenomena more accurately than URANS, but at a much higher computational cost. In addition, in acoustic analogy methods (e.g., the FW-H equation), the inclusion or omission of volume sources (quadrupoles) introduces uncertainty; especially in high-speed regions, neglecting quadrupole sources can lead to under-prediction of high-frequency broadband noise [5].

##### Mesh Resolution and Boundary Conditions

Mesh-independence verification is a key step in reducing numerical error [87]. Mo et al. [88] stressed that the boundary-layer mesh should achieve dimensionless wall distance (y^+^) ≈ 1 to reproduce wall shear stress accurately, and that the acoustic-mesh size must satisfy the λ/6 criterion (λ being the minimum wavelength); otherwise, high-frequency noise resolution will be impaired. Boundary-condition settings (such as non-reflecting boundaries) are crucial to simulating sound-wave propagation accurately; improper settings cause spurious reflections that contaminate far-field predictions.

##### Experiment–Simulation Matching Error

Experimental conditions (e.g., wind-tunnel background noise, sensor-position deviations) and modeling simplifications (e.g., neglecting structural vibration, homogenizing material properties) can both produce validation discrepancies [89]. Wan et al. [90] found that assuming a rigid mirror housing overestimates sound-pressure levels by about 3–5 dB, because the sound-transmission effect of the actual plastic shell is insufficiently modeled. Moreover, an inadequate sampling duration (only 8.87 revolutions in Mo et al. [88]) lowers the spectral resolution and hides high-frequency harmonic components.

In summary, validation of numerical simulations must integrate multi-physics experimental data and quantify the impacts of mesh sensitivity, model selection, and boundary-condition settings. Future research should establish a standardized uncertainty quantification framework to enhance predictive reliability.

### 3.3. Advanced Control Techniques for Aerodynamic Noise

As aerodynamic noise becomes the predominant in-cabin contributor at high speed, its control technologies must balance acoustic performance, aerodynamic characteristics, and engineering feasibility. In recent years, advanced control approaches have diversified, spanning passive control, active and semi-active control, biomimetic design, and multi-objective. Passive control reduces acoustic energy at the source and along the propagation path through shape optimization (e.g., streamlined A-pillar and exterior-mirror designs) and the application of sound-absorbing materials; active control applies interventions such as ANC systems and plasma actuators to achieve dynamic noise reduction; biomimetic design imitates biological structures to innovatively modulate sound-wave propagation; and multi-objective optimization integrates fluid-, acoustic-, and structural-dynamics models to achieve concurrent optimization of noise control and aerodynamic performance. Notably, cutting-edge noise-reduction technologies from aerospace and turbomachinery—such as edge serrations and porous-media applications—are gradually migrating into the automotive domain, underscoring the innovation potential of cross-industry technological convergence [57].

#### 3.3.1. Passive Control Technology

##### Shape-Optimization Design

**Figure 4:**
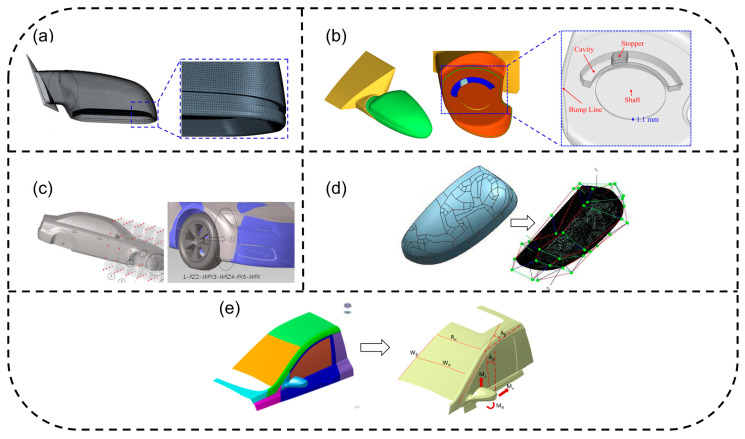
Shape optimization design: (**a**) rectangular cavity structure at mirror edge [91]; (**b**) mirror narrow gap shape optimization [34]; (**c**) fender free deformation optimization [92]; (**d**) mirror housing morphing control [93]; and (**e**) parametric shape optimization using control points and splines [94]. Subfigures (**a**), (**c**–**e**) are under CC BY 4.0 license. (**b**) Reproduced with permission from Lee, Kwongi; et al., *Applied Acoustics*; published by Elsevier Science & Technology Journals (Oxford, UK), 2022; permission conveyed through Copyright Clearance Center, Inc. (New York, NY, USA).

In the source control strategies for vehicle aerodynamic noise, shape optimization design, which suppresses vortex shedding and pressure fluctuations by reconstructing the aerodynamic geometric features of key components, has become a key research focus in recent years. The mirror region, due to its strong separated flow characteristics caused by geometric discontinuity, is the primary optimization target. As shown in Figure 4a, Zhu et al. [91] proposed introducing a rectangular cavity structure at the mirror edge, utilizing fluidic self-excited oscillation characteristics to alter the energy distribution of trailing vortices; experimental validation showed that it significantly reduces mid-to-low frequency noise. Addressing the whistle issue caused by narrow gap flow, Lee et al. [34] employed compressible LES (Figure 4b) to reveal the acoustic feedback mechanism, finding that the coupling effect between the side window geometry and cavity vibrations causes high-frequency noise.

In the field of parametric modeling, the free-form deformation (FFD) technique is widely applied to complex surface optimization due to its efficiency. As depicted in Figure 4c, Jiao et al. [92] established a five-dimensional control parameter model for the fender, combined with a radial basis function (RBF) surrogate model to achieve gradient-driven optimization of aerodynamic noise, demonstrating that synergistic adjustment of the fender leading-edge height and lateral width can weaken the wheelhouse vortex system intensity. Rao et al. [93], as shown in Figure 4d, reconstructed the mirror geometric model based on reverse engineering, identifying through wake field analysis that the asymmetry of the dual-vortex structure on the mirror back surface is the main cause of noise. Multi-parameter collaborative optimization is gradually becoming mainstream. Li et al. [94] (Figure 4e) employed a Latin Hypercube Sampling (LHS) experimental design combined with a genetic algorithm to synchronously optimize the mirror mounting position, A-pillar inclination angle, and windshield curvature, successfully disrupting the interference effect between the mirror wake vortex and the A-pillar separation flow.

In terms of local structural innovation, rectangular cavity structures [91], flow diversion groove designs [95], and edge rounding [96] have proven to be effective for improving flow separation. Chen et al. [97] compared five mirror edge structures through wind tunnel tests, finding that a continuously smooth transition of edge curvature can delay flow separation. The innovative full-perimeter flow diversion groove scheme for the mirror housing proposed by Li et al. [96], combined with optimized inclination angles, significantly improved the interior sound quality.

Current research trends exhibit three key characteristics: first, a shift from isolated component optimization towards integrated vehicle aerodynamic acoustic design; second, the deep application of intelligent algorithms, such as multi-island genetic algorithms and adaptive simulated annealing, enhancing the search efficiency in high-dimensional design spaces; and third, the standard adoption of multi-physics field collaborative verification, where combined simulation frameworks of CAA and SEA significantly improve prediction accuracy.

##### Application of Sound Absorption and Damping Materials

**Figure 5:**
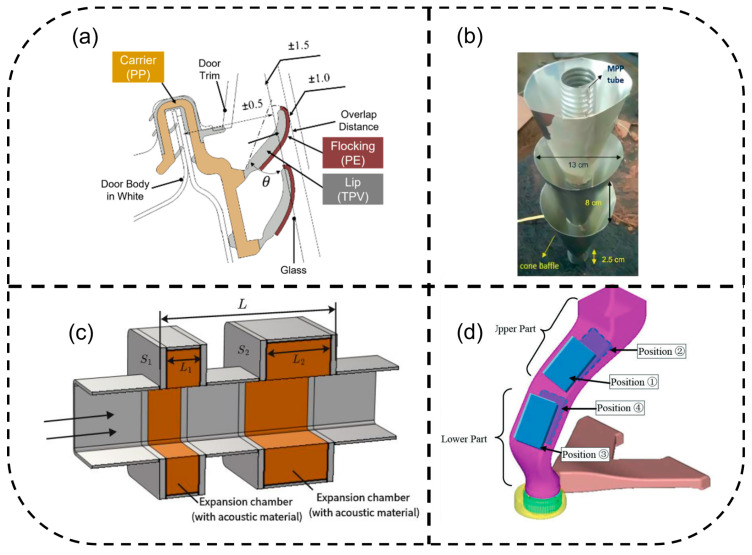
Sound absorption and damping materials: (**a**) schematic illustration of flock-coated TPV door weather-strip [98]; (**b**) MPPs three-cone baffle silencer [99]; (**c**) dual chamber sound-absorbing silencer [101]; (**d**) acoustic cotton placed on the duct [102]. Subfigures (**a**,**c**) are under CC BY 4.0 license. (**b**) Reproduced with permission from Padavala, Prasad; et al., *SAE International Journal of Vehicle Dynamics, Stability, and NVH*; published by SAE International (Warrendale, PA, USA), 2021; permission conveyed through Copyright Clearance Center, Inc. (New York, NY, USA). (**d**) Reproduced with permission from Cao, Yuntao; et al., *SAE International Journal of Engines*; published by SAE International (Warrendale, PA, USA), 2018; permission conveyed through Copyright Clearance Center, Inc. (New York, NY, USA).

In the field of vehicle aerodynamic noise control, innovative applications of sound absorption and damping materials are progressively evolving from passive noise reduction to active–passive collaborative design. Sound-absorbing materials primarily reduce noise propagation by dissipating acoustic energy, while damping materials suppress structural vibrations to block the transmission paths of noise sources. Within airflow channels, materials such as polyurethane foam (PU) and thermoplastic vulcanizate (TPV) are widely used as duct linings, due to their tunable porosity and flow resistance properties. For example, Lee et al. [98] applied a silicone-based fluorescent coating agent to the flocked surface of door seals, as shown in Figure 5a, significantly reducing the friction coefficient at the glass–seal interface and thereby suppressing the generation of high-frequency whistling noise.

For high-frequency noise control, microperforated panels (MPPs) have emerged as a novel solution due to their weather resistance and tunable sound absorption peaks. Regarding low-frequency pulsating noise, a three-cavity MPPs muffler with tapered baffles achieves broadband absorption in the 200–1200 Hz range through partitioned back cavity depths [99], as shown in Figure 5b. In thin-walled structures such as windows, sandwich damping designs demonstrate unique advantages. Hu et al. [100] revealed that the thickness ratio of inner/outer layers in PVB laminated glass differentially affects noise transmission characteristics across frequency bands; optimizing this ratio enables synergistic control over low-frequency turbulence excitation and mid-to-high-frequency acoustic excitation transmission.

Notably, material placement strategies critically influence noise reduction efficacy. Huang et al. [101], as shown in Figure 5c, experimentally confirmed that positioning sound-absorbing materials on the upper duct wall (i.e., near the noise source) enhances noise reduction by approximately 3.6 dB, markedly outperforming other layouts. Furthermore, multifunctional composites represent a new trend in material development. As shown in Figure 5d, Cao et al. [102] implemented a compound design combining cap structures with sound-absorbing liners at battery cooling system intakes, altering noise directivity while achieving an 11 dB(A) intake noise attenuation. Such layered composite structures integrate the sound absorption properties of porous materials with the damping characteristics of the elastic layers, enabling broadband noise control within confined spaces and providing novel approaches for acoustic packaging in future new-energy vehicles.

#### 3.3.2. Active Noise Control Technology

ANC technology counteracts noise by generating sound waves with inverse phase to the original noise, demonstrating significant potential in mitigating vehicle aerodynamic noise. Its core mechanism relies on real-time noise signal acquisition via sensors, controller-generated antiphase sound waves, and actuator output to achieve acoustic interference [103]. In the field of micro electric vehicles, Kato et al. [104] innovatively installed actuators on the front windshield. By directly suppressing interface vibrations, this approach blocked the transmission path of road noise. The solution achieved high-efficiency noise reduction in the low-frequency range (100–500 Hz), with actuator position optimization (e.g., placement at anti-nodes of windshield vibration modes) significantly enhancing control efficacy.

As the technology evolved, Kato’s team [105] further relocated actuators to the roof area and replaced conventional all-pass filters with adaptive filters. This improvement enabled the system to automatically compensate for phase differences between noise and antiphase waves, thereby enhancing performance in single-frequency noise control and laying a critical foundation for tackling broadband noise. Regarding noise classification and mechanism studies, Wang et al. [4] systematically categorized automotive wind noise into three types: sealing leakage noise, body shape noise, and cavity resonance noise. They proposed active control strategies for window buffeting, such as dynamically regulating window gaps or releasing antiphase sound sources. This multi-parameter coordinated method overcomes the limitations of traditional passive control.

To address the broadband and non-stationary characteristics of aerodynamic noise, Wen et al. [3] developed a hybrid aerodynamic active noise control (HAANC) framework. This method integrates variational mode decomposition (VMD) and Hilbert–Huang Transform (HHT) to achieve precise noise feature extraction, while a deep neural network enhances signal fidelity. This significantly improves speech intelligibility and quality in complex acoustic environments. However, widespread commercial implementation faces a fundamental physical bottleneck known as the ‘causality constraint.’ Unlike engine or road noise, which offers coherent reference signals (e.g., RPM or suspension vibration), aerodynamic noise is stochastic, broadband, and lacks a predictive reference source. This makes it difficult for feed-forward algorithms to generate anti-noise before the sound wave reaches the passenger’s ear. Consequently, current ANC efficacy is largely confined to low-frequency cavity booming, while high-frequency broadband wind noise suppression still relies heavily on passive isolation.

#### 3.3.3. Bio-Inspired Design

**Figure 6:**
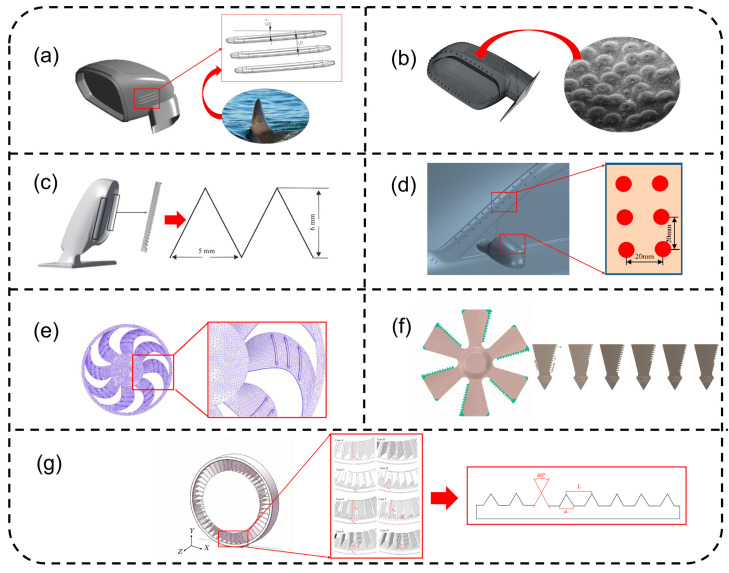
Bio-inspired designs: (**a**) shark-dorsal-fin-inspired mirror design [106]; (**b**) dung-beetle-head-protuberance-inspired mirror design [90]; (**c**) owl-feather-serration-inspired mirror design [107]; (**d**) shell-rib-texture-inspired A-pillar–mirror design [107]; (**e**) biological-surface-contour-inspired fan blade design [47]; (**f**) owl-wing-serration-inspired cooling fan design [45]; and (**g**) shark-skin-riblet-inspired non-pneumatic tire design [36]. Subfigures (**b**,**d**,**g**) are under CC BY 4.0 license. (**a**) Reproduced with permission from Ye, Jia; et al., *Applied Acoustics*; published by Elsevier Science & Technology Journals (Oxford, UK), 2021; permission conveyed through Copyright Clearance Center, Inc. (New York, NY, USA). (**c**) Reproduced with permission from Chen, Xin; et al., *Journal of Hydrodynamics*; published by Springer Nature Customer Service Centre GmbH (Heidelberg, Germany), 2018; reproduced with permission from SNCSC; permission conveyed through Copyright Clearance Center, Inc. (New York, NY, USA). (**e**) Reproduced with permission from Wang, Shuwen; et al., *Proceedings of the Institution of Mechanical Engineers, Part D: Journal of Automobile Engineering*; published by Sage Publications Ltd. (London, UK), 2021; permission conveyed through Copyright Clearance Center, Inc. (New York, NY, USA). (**f**) Reproduced with permission from Hur, Kwang Ho; et al., *International Journal of Aeroacoustics*; published by SAGE Publications (Thousand Oaks, CA, USA), 2023; permission conveyed through Copyright Clearance Center, Inc. (New York, NY, USA).

Bio-inspired morphological modifications have evolved from empirical biomimicry into a systematic strategy for passive aeroacoustic control, primarily by manipulating boundary layer development and vortex shedding dynamics. As illustrated in Figure 6, these designs are categorized by their underlying fluid–structure interaction (FSI) mechanisms. The first category focuses on boundary layer modulation and pressure field homogenization. Ye et al. [106] (Figure 6a) utilized shark-fin-inspired riblets to “comb” surface streamlines on side mirrors, which suppresses cross-flow instabilities and reduces the negative-pressure gradient in the wake. This mechanism achieves a significant aerodynamic synergy, where the suppression of turbulent kinetic energy (TKE) is accompanied by a reduction in pressure drag. Similarly, Wan et al. [90] (Figure 6b) and Liu et al. [107] (Figure 6d) employed hemispherical convex arrays, inspired by beetle-head protuberances and shell textures, to disrupt large-scale vortex coherence. In both cases, the bionic structures act as micro-vortex generators that stabilize the boundary layer; notably, Liu et al. [107] reported that this reorganization of the A-pillar and mirror wake concurrently lowers both the drag ($C_{d}$) and lift ($C_{l}$) coefficients, thereby enhancing vehicle stability at high speeds.

The second core mechanism involves vortex fragmentation and phase interference, which are typically achieved through edge treatments or non-smooth textures. Inspired by the silent flight of owls, Chen et al. [108] (Figure 6c) implemented trailing-edge (TE) serrations on mirror housings to fragment large-scale shear layer vortices into smaller, incoherent structures. Unlike surface protrusions, these edge treatments generally maintain a neutral aerodynamic trade-off, as they focus on high-frequency noise suppression (500–5000 Hz) without significantly altering the mirror’s frontal area or primary pressure distribution. In the specialized domain of non-pneumatic tires (NPT), Zhou et al. [36] (Figure 6g) demonstrated that riblet-textured spokes suppress the Lamb vector intensity within the open flexible-spoke structure. The acoustic benefit of 5.18 dB is achieved with minimal impact on rolling resistance, provided the riblet orientation is optimized for the local flow angle, representing a highly localized form of vortex control.

For rotating components and dynamic surfaces, however, the implementation of bionic features often encounters a performance trade-off. Wang et al. [47] (Figure 6e) found that while bionic ridge-like textures on cooling fan blades can delay the laminar-to-turbulent transition to reduce noise by 3.83 dB(A), the added wetted surface area can increase skin friction drag if the ridge spacing is not perfectly aligned with the Reynolds number. This efficiency penalty is even more pronounced in the work of Hur et al. [45] (Figure 6f), who investigated TE serrations on axial fans. Despite a substantial noise reduction of 11 dB via phase interference, the reduction in the effective blade area leads to a measurable drop in the volumetric flow rate and static efficiency. To maintain identical cooling performance, the fan must operate at higher rotational speeds, which may partially offset the initial acoustic gains. These findings emphasize that for dynamic bionic applications, the optimization of the noise–drag–efficiency triplet is essential for practical viability. A systematic comparison of the aforementioned bio-inspired strategies, encompassing their biological prototypes, underlying physical mechanisms, and quantitative noise reduction performance, is consolidated in Table A1 (see Appendix A).

Despite the theoretical efficacy demonstrated in numerical and wind-tunnel studies, the industrialization of bionic aeroacoustic structures faces critical hurdles related to manufacturing fidelity, durability, and maintenance. High-fidelity micro-features, such as the 5mm riblets in [106] or the fine serrations in [107], require high-precision injection molding that significantly increases tooling costs and production cycle times. Furthermore, in real-world road environments, these micro-structures are highly susceptible to “surface fouling.” The accumulation of dust, road salt, or ice within riblets or between serrations can fundamentally alter the surface roughness, potentially converting a noise-reduction feature into a source of parasitic turbulence. Moreover, standard maintenance procedures like high-pressure washing can cause mechanical wear on delicate bionic edges, leading to acoustic performance degradation over the vehicle’s lifecycle. Future research must prioritize “Robust Bionics”—designing structures that maintain functional integrity despite environmental contamination and manufacturing tolerances.

### 3.4. Design Methodologies and Optimization Strategies

Synergistic optimization for vehicle aerodynamic noise requires balancing conflicting objectives of acoustic performance, aerodynamic efficiency, and engineering constraints [109]. Multi-objective approaches achieve comprehensive breakthroughs through intelligent algorithms and cross-disciplinary integration. Guo et al. [110] proposed a phased optimization strategy for fuel-cell vehicle high-voltage cooling fan systems: First, parametric modeling adjusted the blade installation angles and outer ring structures to reduce single-fan noise. Subsequently, the response surface methodology (RSM) established a spatial parameter and flow-noise response model for dual-fan layouts. An entropy-weighted evaluation function optimized the system configuration, ultimately achieving a 12.8% flow increase while reducing noise by 0.98 dB. This study quantitatively revealed the nonlinear impact of the radiator distance, fan axial spacing, and other parameters on acoustic performance.

In sealing system design, Lee et al. [98] achieved revolutionary breakthroughs via TPV material modification. Increasing the ethylene–propylene rubber crosslink density reduced the compression set to 27.6% and raised the loss modulus to 323 MPa for enhanced damping. Concurrently, silicone-based fluorescent coating reduced the friction coefficient to 0.30. This material–structure–surface co-design lowered door seal wind noise by 5 dB(A) while eliminating friction-induced squeal. Zhang et al. [5] quantified seal sound transmission mechanisms using SEA, establishing transmission loss models (e.g., side window seals: TL = 20 dB at 4000 Hz). They introduced a cavity acoustic coupling model revealing the door-gap cavity’s dominant contribution below 1600 Hz, providing quantitative guidance for early-stage design.

Optimization algorithm innovations significantly improved computational efficiency. Li et al. [94] employed genetic algorithms to co-optimize eight mirror-region parameters. Subdomain simulation reduced computational costs to 4% of the full-model requirements. Acoustic power contribution analysis (side windows: 70.3%) identified critical noise paths, ultimately reducing the driver ear sound pressure level by 2.08 dB(A). Beigmoradi et al. [39] utilized fractional factorial design for dimensionality reduction in hatchback rear-end optimization. A 16-simulation regression model analyzed the parameter sensitivity, revealing the spoiler angle’s 38% contribution to the drag coefficient and its 25% interaction effect with the diffuser angle on aerodynamic noise, establishing an efficient engineering iteration framework.

The current research is evolving toward dynamic control systems: developing adaptive airflow control devices (e.g., active micro-jets) based on vortex evolution characteristics, or constructing real-time noise source-transfer path mapping models via machine learning, advancing from static optimization to closed-loop control.

## 4. Conclusions

This review synthesizes significant advancements in vehicle aerodynamic noise management, driven by the imperative to enhance NVH performance in electrified and autonomous vehicles. Mechanistically, research has quantitatively decoupled the spatiotemporal evolution of core noise sources—A-pillar conical vortices, mirror wake vortices, underbody separation flows, and wake interactions—through wavenumber-frequency decomposition and dynamic NTF. Numerically, hybrid simulation frameworks (e.g., LES/FW-H, LBM/SEA) have matured, balancing accuracy and efficiency via standardized mesh optimization (y^+^ ≤ 1, λ/6 criterion) and non-reflecting boundary conditions. Control technologies now span passive strategies (streamlined A-pillars, micro-perforated panels), active systems (Terfenol-D actuators, adaptive ANC), and bio-inspired designs (shark-fin riblets, owl-wing serrations), collectively enabling synergistic noise–drag trade-offs through multi-objective optimization. These integrated advances provide a robust foundation for next-generation vehicle acoustic refinement.

Future research must urgently tackle multifaceted challenges. Mechanism exploration requires deeper investigation into transient seal nonlinearity, high-frequency sound transmission in lightweight composites, and psychoacoustic perception shifts in autonomous cabins. Simulation innovation should prioritize real-time fluid–structure–acoustic full-coupling platforms, Lattice Boltzmann-based broadband resolution, and digital twin environments embedding uncertainty quantification. Intelligent control demands AI-driven active flow modulation (plasma/synthetic jets), self-adaptive metamaterials for low-frequency suppression, and reinforcement learning for dynamic noise–drag–thermal co-optimization. Concurrently, human-centric frameworks must evolve to quantify auditory fatigue in non-driving scenarios and extreme conditions (crosswinds/tunnels). Cross-industry technology migration (e.g., aerospace vortex control) and standardized validation protocols will accelerate solutions, ultimately enabling silent, efficient mobility for the autonomous era. Another critical challenge lies in the discrepancy between idealized laboratory conditions and complex real-road environments. Current CFD simulations and wind tunnel tests predominantly utilize steady, uniform inflow and dry conditions, which fail to account for the transient effects of fluctuating crosswinds and diverse weather phenomena (e.g., precipitation and humidity). These environmental variables can significantly alter the boundary-layer characteristics and vortex stability, potentially degrading the performance of finely tuned bionic structures. Future research should therefore transition toward “all-weather” aeroacoustic modeling to bridge the gap between theoretical optimization and practical engineering reliability.

## Figures and Tables

**Table 1:** Inclusion and exclusion criteria applied during the screening of studies for this systematic review, specifying the publication scope, technical relevance, and vehicle applicability.

Criterion	Inclusion	Exclusion
Time span	1 January 2016–20 June 2025	Outside range
Document type	Peer-reviewed journal article	Conference paper, thesis, patent, report
Content	Numerical aeroacoustic simulation (CFD, LES, CAA, BEM, hybrid) and/or advanced mitigation technology (ANC, plasma actuation, acoustic metamaterials, flow-control devices, shape optimization)	Studies focusing solely on steady-state aerodynamic drag without acoustic pressure analysis; research lacking high-fidelity numerical resolution (e.g., LES, DES, LBM) or specific noise mitigation frameworks
Vehicle scope	Road vehicles: passenger cars, trucks, buses	Aircraft, trains, motorcycles, UAVs, eVTOL

**Table 2:** Ideal aerodynamic source types and their key characteristics for road-vehicle noise [20,21,22].

Source	Ideal Model	Mechanism	Locations	Relationship Between I and v
Monopole	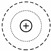	Periodic volumetric pulsation/suction	Door-seal gaps, body joints	$I_{\mathrm{monopole}}\;\sim \;v^{4}$
Dipole	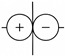	Unsteady wall-pressure forces	A-pillar, exterior mirror, wheel arch	$I_{\mathrm{dipole}}\;\sim \;v^{6}$
Quadrupole	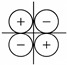	Turbulent shear-stress fluctuations	Wake, underbody shear layers	$I_{\mathrm{quadrupole}}\;\sim \;v^{8}$

**Table 3:** Core metrics for stationary wind-noise perception (adapted from Carr et al. [50]).

Metric	Symbol/Unit	Domain	Main Aspect Captured	Correlation with Annoyance
A-weighted SPL	LAeq (dB A)	Physical	Overall sound-pressure level (baseline reference)	$\mathrm{Moderate}\;(R^{2}\;\approx \;0.65$); alone cannot fully explain discomfort
Zwicker loudness	N (sone)	Psycho-acoustic	Perceived volume after auditory weighting	$\mathrm{Strongest}\;\sin\mathrm{gle}\;\mathrm{predictor}\;(R^{2}\;\approx \;0.79$)
Sharpness	S (acum)	Psycho-acoustic	High-frequency spectral balance (“shrillness”)	Adds ≈ 10% explanatory power when combined with loudness
Roughness	R (asper)	Psycho-acoustic	Fast (20–300 Hz) amplitude modulation, felt as “harshness”	Small but significant influence in broad-band cases
Subjective annoyance score	1–9 pt jury scale	Subjective	Overall occupant discomfort	Target ≤ 5 pts for acceptable cabin quality

**Table 4:** Mainstream numerical simulation methods of aerodynamic noise.

Method	Workflow	Pros	Cons	Ref.
DNS	Solve full comp. Navier–Stokes; sound direct	Highest fidelity	$\mathrm{Only}\;\mathrm{low}\;R_{e}$; huge cost	[60]
LES + FW-H	LES field → FW-H radiation	Captures broadband	Fine grid, still costly	[61]
DES/DDES + FW-H	RANS/LES mix → FW-H	Good accuracy–cost compromise	Under-resolves very small eddies	[62]
URANS + Curle analogy	URANS flow → Curle dipoles	Fast, robust screening of low-frequency tonal noise	Broadband/high-frequency noise poorly captured	[63]
LBM	Compressible LBM; flow and sound together	Scales well on GPUs; good mid–high-frequency resolution	Large lattices at high Re; stability and BC tuning needed; modern formulations support high-Ma flows.	[64]

## Data Availability

The data presented in this study are available within the article and its supplementary materials.

## Supplementary Materials

The following are available online at https://www.mdpi.com/article/10.3390/biomimetics11020099/s1, Table S1: PRISMA checklist.

## Abbreviations

The following abbreviations are used in this manuscript:

ANC	Active Noise Control
APE	Acoustic Perturbation Equation
ATPA	Acoustic Transfer Path Analysis
BEM	Boundary Element Method
CAA	Computational Aeroacoustics
CFD	Computational Fluid Dynamics
DES	Detached Eddy Simulation
DDES	Delayed Detached Eddy Simulation
DNC	Direct Numerical Computation
FFD	Free-Form Deformation
FW-H	Ffowcs Williams–Hawkings
HAANC	Hybrid Aerodynamic Active Noise Control
HHT	Hilbert–Huang Transform
ICA	Independent Component Analysis
IDDES	Improved Delayed Detached Eddy Simulation
LAA	Lighthill Acoustic Analogy
LBM	Lattice Boltzmann Method
LES	Large Eddy Simulation
LHS	Latin Hypercube Sampling
MPPs	Microperforated Panels
NPT	Non-Pneumatic Tire
NTF	Noise Transfer Function
NVH	Noise, Vibration, and Harshness
PU	Polyurethane Foam
RANS	Reynolds-Averaged Navier–Stokes
RBF	Radial Basis Function
RSM	Response Surface Methodology
SBES	Stress-Blended Eddy Simulation
SEA	Statistical Energy Analysis
SPL	Sound Pressure Level
TL	Transmission Loss
TPV	Thermoplastic Vulcanizate
URANS	Unsteady Reynolds-Averaged Navier–Stokes
VMD	Variational Mode Decomposition
y^+^	Dimensionless Wall Distance

## Appendix A

Main characteristics of the studies included in the systematic review.

**Table A1:** Summary of bio-inspired strategies for vehicle aerodynamic noise control.

Study	Bionic Prototype	Application	Key Mechanism	Method	Performance
Ye et al. (2021) [106]	Shark dorsal fin	Mirror surface	Reduces negative pressure and TKE	Hybrid CAA	7.3 dB reduction
Wan and Ma (2017) [90]	Beetle head bumps	Mirror housing	Suppresses vortex pair formation	CFD and Tunnel	10 dB reduction
Chen et al. (2018) [107]	Bionic serrations	Mirror edges	Weakens vortex sound coupling	Exp. and CFD	500 Hz or higher suppression
Liu et al. (2018) [108]	Shell ribs	A-pillar and window	Reorganizes horseshoe vortices	Transient CFD	20 dB reduction
Wang et al. (2021) [47]	Ribbed surface	Cooling fan blades	Delays transition and minimizes secondary vortices	Exp. and Orthogonal	3.83 dB(A) reduction
Hur et al. (2023) [45]	Owl wing serrations	Cooling fans	Disrupts trailing edge coherence	LES and LAA	10 dB reduction
Zhou et al. (2020) [36]	Shark skin riblets	Non-pneumatic tire	Fragments vortices and reduces strain energy	LES	5.18 dB reduction

**Table A2:** Summary of aeroacoustic background and generation mechanisms.

Study	Region	Primary Mechanism	Methodology	Key Findings
Hu et al. (2021) [27]	Side Window	Helmholtz resonance and vortex shedding	CFD and CAA	Deflectors reduce buffeting by disrupting vortex paths
Ali et al. (2018) [28]	A-pillar and Mirror	Conical vortex formation and wake interaction	CFD (DriAver)	Mirror turbulence dominates high frequency noise
Lee et al. (2022) [34]	Mirror Gap	Acoustic feedback loop and gap flow instability	Compressible LES	Gap geometry triggers narrow band tonal whistling
Chode et al. (2023) [40]	Mirror and Wake	Horseshoe and A-pillar vortex interference	Hybrid CAA	16 degree tilt reduces noise by 10 dB
Uhl et al. (2023) [68]	Mirror Region	Turbulent wall pressure fluctuations	CAA and Stochastic	Efficiently predicts broadband noise
Li et al. (2018) [94]	Mirror and A-pillar	Interaction between wake and separation flow	GA and CFD	Optimization reduces driver ear noise by 2.08 dB(A)

**Table A3:** Summary of numerical methodologies and EV NVH challenges.

Study	Research Object	Core Methodology	Physical Model	Key Metrics	Main Contribution
Chen et al. (2024) [58]	Passenger Vehicle	Error Source Analysis	SAS and LRN k−ε	$C_{d}$	Standardized CFD simulation strategy
Guo et al. (2022) [109]	FCV Cooling Fan	RSM and Box–Behnken	LES and FW-H Analogy	SPL and Flow Rate	Aeroacoustic optimization of FCV fans
Hamiga et al. (2020) [79]	Ahmed Body (25°)	CFD-CAA Coupling	k−ω SST and LES	Drag and Flow Topology	Baseline model for moving vehicle noise
Hua et al. (2021) [52]	BEV NVH	Systematic Review	BEM and FEM	Tonal Noise	Defined NVH challenges in engine-less EVs
Kato et al. (2016) [104]	Micro-EV Window	Active Noise Control	Magnetostrictive Model	Vibration and dB	Structural excitation for road noise control
Beigmoradi et al. (2021) [39]	Hatchback Rear	Fractional Factorial	Realizable k−ε	$C_{d}$ and Acoustic Power	Multi-objective geometry optimization
Cavaliere et al. (2023) [11]	BiW Structure	ROM and PGD Algorithm	Parametric FEA	Modal and Stiffness	Real-time NVH visualization and solver
Li et al. (2017) [83]	Intake System	LES-FEM Coupling	k−ε and LES	Transmission Loss	Validated LES-FEM for duct acoustics
Ma et al. (2025) [6]	Cabin Wind Noise	WOA-Xception (AI)	Shape-Feature CNN	Loudness and MAE	Rapid prediction based on body shape
Moron et al. (2023) [72]	On-Road Turbulence	CFD-SEA Hybrid	LBM (VLES)	Modulation Noise	Real-world vs. wind tunnel discrepancies
Münder et al. (2022) [2]	EV Soundscape	Perceptual Analysis	Psychoacoustic Metrics	Annoyance and Sharpness	Perceptual standard for BEV evaluation
Kato et al. (2018) [105]	Micro-EV Cabin	Small Actuator ANC	Feedback Control	Noise Gain (dB)	Compact ANC for space-limited EVs
Li et al. (2022) [96]	SUV Full-Scale	APE-SEA Method	APE and k−ε	AI and SPL	High-frequency wind noise optimization
Oettle et al. (2019) [78]	Door and Window Seals	LBM-SEA Hybrid	Transmission Loss	Interior SPL	Seal performance prediction in early design
Oettle et al. (2017) [1]	Automotive CAA	Technology Overview	Source-Path Receiver	Drag and Aero-Noise	Evolution of aeroacoustic design trends
Padavala et al. (2021) [99]	Pure BEV	EMA and Masking Tests	Experimental Modal	Order Tracking	Identified electric powertrain tonal issues
Qian et al. (2021) [12]	EV Sound Quality	SA-GA-BPNN (AI)	Objective–Subjective Map	Psychoacoustic Index	Intelligent evaluation of BEV sound quality
Wen et al. (2025) [3]	Cabin Voice	Transformer-based AI	Time–Frequency Hybrid	SNR and Signal Loss	Wideband noise reduction and voice recovery
Yao et al. (2019) [74]	Glass Window	LES-Vibro-Acoustics	Fluid–Structure (FSI)	Wavenumber-Freq	Characterized turbulence-induced vibration
Zhan et al. (2021) [13]	Poroelastic Media	Freq-Domain SEM	Anisotropic Media	Wave Attenuation	Efficient modeling for cabin trim materials
Zhang et al. (2025) [5]	SUV Full-Scale	SEA	Leakage and Path	Articulation Index	Quantified seal impact on cabin wind noise

**Table A4:** Summary of others.

Study	Research Object	Key Findings (Quantitative/Qualitative)
Wang et al. (2021) [4]	Vehicle Body	Qualitative: Wind noise contribution increases with speed; difficult to control side window buffeting passively
Horváth et al. (2024) [7]	Electric Vehicle Powertrain	Qualitative: System-level approach required for NVH management in EVs
Deng et al. (2023) [8]	Electric Vehicle Chassis	Qualitative: Battery pack affects flow/pressure fields; drag/lift coefficients increase
He et al. (2020) [25]	DrivAer Side Glass	Qualitative: Acoustic pressure fluctuations have higher transmission efficiency than convective; acoustic fraction dominates above coincidence frequency
Hou et al. (2021) [26]	Vehicle Wind Tunnel	Qualitative: Observations from wind tunnel measurements for wind noise development
Azman et al. (2024) [29]	Vehicle Side Mirror	Quantitative: Horizontal base produces 103.41 dB at 120 km/h; Angular base produces 101.48 dB at 120 km/h
Hao et al. (2022) [30]	Electric Vehicle Rearview Mirror	Qualitative: Electric vehicle shows less turbulent pressure and sound pressure levels
Jamaludin et al. (2023) [31]	Automotive Side Mirror	Quantitative: Sedan mirror produces 77.21 dB at 120 km/h; SUV mirror produces 75.71 dB at 120 km/h
Zaareer et al. (2022) [32]	Vehicle Side Mirror	Qualitative: Horizontal base generates noticeably higher noise than angular placement
Yuan et al. (2017) [33]	Rear View Mirror	Qualitative: Flow field around mirror is 3D, unsteady, separated, and turbulent
Dinh et al. (2022) [35]	Car Side Mirror	Qualitative: Analyzes turbulent flow structure and predicts external acoustic field
Yang et al. (2024) [37]	Electric Vehicle Underbody	Qualitative: Underbody airflow increasingly affects interior noise; evaluated side skirts and wind deflectors
Wang et al. (2021) [38]	Vehicle Underbody	Qualitative: Underbody contributes mainly to low/middle frequencies; investigated panel thickness effects
Saf et al. (2020) [41]	Vehicle Door Seals	Qualitative: STL affected by material, cross-section design, and system dynamics
Sun et al. (2022) [42]	Centrifugal Air Compressor	Quantitative: Noise reduced by 4.1 dBA (structure); 5.8 dBA (muffler); total reduction from 78.8 to 68.9 dBA
Hua et al. (2017) [43]	Automobile Alternator	Quantitative: Average noise level decreased by 2.58 dB; mass flow increased by 1.36 g/s
Miyamoto et al. (2017) [44]	Automobile Bonnet	Qualitative: Tonal noise effectively reduced; separation around kink suppressed by PA control
Ren et al. (2023) [46]	Automotive Cooling Fan	Qualitative: Main noise at tip of forward swept wing; SPL metrics analyzed
He et al. (2021) [48]	DrivAer Side Window	Qualitative: Hydrodynamic pressure loses more energy than acoustic; side window acts as low-wavenumber filter
Fukushima et al. (2016) [49]	Vehicle Body	Qualitative: New transmission model treats sources as forces; quantitative synthesis of interior noise
Carr et al. (2021) [50]	Vehicle Interior	Qualitative: Models improved by including sharpness metric with loudness
Carr et al. (2022) [51]	Vehicle Interior	Qualitative: Gusting metric needed for non-stationary wind noise acceptability
He et al. (2018) [53]	DrivAer Front Side Window	Qualitative: Good agreement of up to 1000 Hz between calculated and measured radiation
Talay et al. (2019) [55]	Vehicle Door	Qualitative: Door stiffness and sealing gap affect interior wind noise at high speeds
Yin et al. (2019) [56]	Automobile Side Window	Qualitative: SPL and loudness increase with velocity; sharpness decreases with window opening degree
Yadegari et al. (2020) [57]	Multiple: Side mirrors, A-pillars, Vehicle body	Qualitative: Reviewed noise reduction techniques across aerospace, turbomachinery, and automotive industries
Masri et al. (2024) [59]	Electric/Hybrid Vehicle	Qualitative: NVH sources shift from powertrain (ICE) to road-tire and wind-structure interactions at high speeds
Zhu et al. (2017) [62]	High-speed Train	Qualitative: Main noise from leading bogie; inter-carriage gap causes tonal noise; peak A-weighted SPL at ~1 kHz
Li et al. (2019) [64]	Computational Aeroacoustics	Qualitative: LBM shows superior space–time resolution for direct/indirect noise computations
Duan (2020) [66]	SUV Rear-view Mirror	Quantitative: Interior SPL reduced by 6.41%; speech intelligibility improved by 33.89%
Guseva et al. (2022) [67]	Generic Side Mirror	Qualitative: Validated hybrid simulation method; good agreement with experimental data
Ali et al. (2018) [69]	Generic Vehicle	Qualitative: Good SPL agreement (200-2000 Hz) between calculation and experiment
Zhong et al. (2019) [70]	Passenger Vehicle	Qualitative: Major sources: underbody (<200 Hz), windows (>200 Hz); 260 M cells give better accuracy
Dawi et al. (2019) [71]	Generic Vehicle Model	Qualitative: Demonstrated direct noise computation; compared with/without side mirror
Liang et al. (2020) [75]	Harvester vehicles	Qualitative: Flow field homogenization and vortex reconstruction
Liang et al. (2020) [73]	Rice Combine Harvester Fan	Quantitative: Requested airflow 3.0 m^3^/s; upper duct 8–9 m/s; middle section 4–6 m/s; tail section 3–4 m/s
Tajima et al. (2024) [76]	Vehicle in Wind Conditions	Qualitative: Fluctuating wind noise is amplitude-modulated aerodynamic noise; MPS analysis enables quantitative evaluation
Ding et al. (2022) [77]	Rice Combine Harvester	Qualitative: Grain sieve losses and impurity ratio improved dramatically with multi-duct cleaning
Xu et al. (2020) [80]	Rice Combine Harvester	Quantitative: Prediction error < 9.4% for cleaning loss ratio; < 11.7% for grain impurity ratio
Deng et al. (2018) [82]	Automotive Door Sealing	Qualitative: TL optimization using orthogonal design based on articulation index
Wang et al. (2017) [84]	Vehicle Rear Window	Quantitative: SPL calculation error <2%; Both sides open much quieter than single window
Tang et al. (2017) [85]	Rotating Drive Component	Qualitative: Unsteady fluid impact loading and surface stress distribution optimization
Broatch et al. (2016) [86]	Automotive Turbocharger	Qualitative: Noise increases toward surge; high-frequency stall oscillations; whoosh noise from rotating cells
Fordjour et al. (2020) [87]	Rotating Drive Component	Qualitative: Enhanced acoustic stability via precise rotational
Mo et al. (2020) [88]	Automotive Cooling Fan	Quantitative: Tonal noise 110 dB SPL at blade tip; 5–6 dB decay per distance doubling
Hu et al. (2021) [89]	Vehicle Thermal Management Fan	Qualitative: Significant suppression of turbulent kinetic energy and flow-induced acoustic sources.
Zhu et al. (2018) [91]	Rear View Mirror	Quantitative: Maximum noise decrease rate 15.62%; minimum 8.90%
Jiao et al. (2024) [92]	Passenger Car Fender	Qualitative: Fender shape optimization reduces aerodynamic noise; investigated flow field characteristics
Rao et al. (2018) [93]	Rearview Mirror	Quantitative: Maximum noise reduction amplitude up to 3 dB after optimization
Rao et al. (2017) [95]	SUV Rearview Mirror	Qualitative: Analyzed flow field and noise characteristics behind mirror using reconstructed 3D model
Lee et al. (2023) [98]	EV Door Weatherstrip	Quantitative: Wind noise reduced 5 dB(A); friction coefficient reduced 80%
Hu et al. (2024) [100]	Rearview Mirror and Side Window	Qualitative: Mirror body proportion, lower edge angle, column length affect noise in order
Huang et al. (2024) [101]	Electric Vehicle AC System	Qualitative: Addressed non-planar wave cavity resonance in EV air conditioning noise
Cao et al. (2018) [102]	HEV Battery Cooling	Qualitative: Cap structure modified noise directionality; fan speed optimized based on masking
Shen et al. (2024) [103]	Combine Harvester	Quantitative: Detection error 6.1% between detected and actual loss amounts
Liang et al. (2022) [109]	Harvester vehicles	Qualitative: Flow field consistency and noise source suppression achieved through dual fan cooperation
